# The effect of breast cancer awareness interventions on young women aged 18–50 years: A systematic review

**DOI:** 10.1177/13591053241270614

**Published:** 2024-08-12

**Authors:** Caitríona Plunkett, Melissa Pilkington, Joseph Keenan

**Affiliations:** Manchester Metropolitan University, UK

**Keywords:** breast cancer, health behaviour, intervention, systematic review, young women

## Abstract

A scarcity of research has examined the effect of breast cancer awareness (BCA) interventions among young women (18–50 years). This overlooks important differences that may affect BCA levels such as education preferences within this younger cohort. Younger women are more likely than older women to present with aggressive subtypes of breast cancer if they develop the disease, and at a more advanced stage translating into poorer survival. It is therefore worthy to investigate which interventions have a significantly positive effect on BCA within this cohort. Five studies were deemed eligible for review. Despite differing intervention methods, theoretical applications and awareness targets, positive outcomes were reported across all designs. However, the evidence is weak in investigating the effectiveness of BCA interventions on this cohort and is considered as inconclusive with such a small number of available studies to review, highlighting a need for further research in this area.

## Rationale

While there is substantial literature available concerning breast cancer awareness (BCA), much of it explores individuals’ attitudes towards specific components of BCA such as screening (e.g. Eibich and Goldzahl, 2020), and breast self-examination (e.g. Nde et al., 2015), or it aims to examine BCA levels of particular cohorts such as within communities (e.g. Chattu et al., 2018 (Buraimi, Oman); Dey et al., 2015 (Delhi, India); Mena et al., 2014 (rural Ghana)). Considerably less of this directly measures several outcomes simultaneously of BCA. Furthermore, there is a prevalence of this literature being aimed at older women that are over 50 years of age (e.g. Forbes et al., 2011; Linsell et al., 2008), or at *all* and *any* women (Laughman et al., 2017; Taha et al., 2014; Talib et al., 2016). Despite nearly a quarter of breast cancer cases occurring before 50 years of age (Cardoso et al., 2019), a dearth of research has been conducted to examine BCA within this cohort. This omission overlooks important differences that may affect BCA levels such as education preferences.

Younger women are more likely than older women to present with aggressive subtypes of breast cancer if they develop the disease, and at a more advanced stage translating into poorer survival rates (Cathcart-Rake et al., 2021). High-risk features include oestrogen receptor negative or HER2 subtypes with a high grade 3 histology and lymphatic penetration (Parker et al., 2009). It is therefore important to investigate which interventions have a significantly positive effect on BCA among young women who are aged under 50 years.

Within Almutairi et al.’s (2019) systematic review conducted to examine knowledge and awareness of breast cancer and risk factors among young women (age range across studies of 14–52 years), the importance of BCA within this cohort is highlighted to aid in lowering breast cancer mortalities. The importance of detecting breast cancer early by increasing BCA therefore cannot be overstated. However, BCA appears to be multi-faceted and variable within its components of what is understood as BCA, including terms that have been carried over owing to historical influences such as breast self-examination (BSE) which traditionally followed repetitive palpations of a formally taught set method at the same time each month to detect any breast changes (Thornton and Pillarisetti, 2008). However, within contemporary societies, particularly those with more advanced healthcare systems, there is a reduction in BSE utilised as a stand-alone intervention. Rather, it has become incorporated as a BCA component alongside screening and risk factor education, and understood more broadly as awareness instead of a rigorously set breast examination. To the best of the researchers’ knowledge, no recent systematic reviews investigating BCA interventions have included BSE as a search term (e.g. Anastasi and Lusher, 2019; O’Mahony et al., 2017).

## Objectives

The objective of this review was to assess the effect of BCA interventions on young women’s (aged 18–50 years) BCA knowledge levels compared to standard available care, or no intervention.

## Methods

### Research design

This systematic review is conducted in accordance with the Preferred Reporting Items for Systematic Reviews and Meta-Analysis (PRISMA) checklist. Methods of the analysis and inclusion criteria were neither specified in advance, nor documented in a protocol.

## Eligibility criteria for review

### Studies

Experimental design (randomised controlled trials (RCTs), or controlled clinical trials (CCTs)), or quasi-experimental design of pre-test and post-test design studies that examine the effect of BCA interventions on young women’s (aged 18–50 years) BCA knowledge levels were considered. Studies published in their entirety in English, within the period of 2012–2022, and with an outcome of BCA knowledge of young women were eligible for inclusion.

Journals from 2012 onwards have been included to reflect a move away from BSE method as a rigorously followed technique that has been attributed to psychological distress particularly among hypervigilant individuals (van Dooren et al., 2003). Instead, BSE within contemporary foci of highlighting awareness of what is normal for the individual is adopted.

### Participants

Women aged between 18 and 50 years (inclusive) were specifically targeted. Studies with participants not within this age range, or that did not communicate age ranges were excluded. No restrictions were placed on the setting of where individuals were recruited.

### Intervention

Studies comparing the effect of BCA interventions on young women’s (aged 18–50 years) BCA knowledge levels with standard available care or no intervention comparison were included.

### Outcome measure

Studies that included either primary or secondary outcome measures of BCA knowledge levels were regarded as the principal outcome measure. Self-reports by participants, and/or use of any measurements that measure components of BCA were included.

## Search methods for identification of studies

Seven databases were searched by the Principal Investigator (PI) to identify studies of interest of MEDLINE through PubMed, Cochrane Library (CENTRAL), APA PsycArticles, APA PsycInfo, ASSIA, CINAHL and Web of Science. It is acknowledged that database selection should be directed by the review topic (Lorenzetti et al., 2014). Web of Science is recognised as the world’s leading scientific citation search and analytical information platform (Li et al., 2018). MEDLINE is recommended by Cochrane for its broad wide-ranging database, consisting of approximately 30 million references to journal articles pertaining to health matters (Lefebvre et al., 2024). With BCA interventions also researched by nursing and allied health professionals, CINAHL, accessed through EBSCOhost, was also included. Both APA PsycArticles and APAPsychInfo through EBSCOhost were included as they provide information on studies and interventions such as that within BCA behaviour. While APA PsycArticles provides full-text and peer reviewed articles from top-cited psychology journals, APA PsycInfo only provides abstracts and index resources (APA, 2024). This is similar to ASSIA (accessed through ProQuest), but it was deemed necessary to include this database due to its inclusion of pertinent areas within this research including health, psychology and education (ProQuest, 2022). Cochrane’s Central Register of Controlled Trials (CENTRAL) also does not contain full article texts, but it was included to identify randomised and quasi-controlled trials (Cochrane Library, 2022). Access to the papers’ full texts were subsequently found within other included databases such as CINAHL, and PubMed and through Manchester Metropolitan University’s online library.

Only research in English was reviewed due to not having translation services. No missing information such as study methods or results was acquired from investigators or sponsors. Instead, any research that demonstrated these omissions were excluded. The search was run from 2nd to 9th November 2022, inclusive.

There were slight variations in limiter terms depending on the database (Supplement 1) to align with eligibility criteria. However, search terms and Boolean phrases remained constant:

‘breast cancer aware*’ OR ‘breast aware*’ OR ‘breast self-exam*’ OR ‘breast self exam*’ AND intervention OR program OR programme OR educat* OR promot* AND woman OR women OR ‘young woman’ OR ‘young women’ OR student

With MESH terms available for MEDLINE, these were also utilised to widen the inclusion of potential literature suitable for the systematic review (Supplement 2). However, an extensive search of terminology variation and phrases through literature and previous systematic reviews was conducted prior to final determination of search terms and Boolean phrases employed, with the * function utilised to broaden possible stem variations of words within database searches.

## Study selection

Each database returned the following results for review: Web of Science (333), CINAHL (165), ASSIA (138), APA Psychinfo (101), MEDLINE (33), Cochrane Library (CENTRAL; 23), APA PsychArticles (0).

## Data extraction

Information accuracy was not verified with the primary researchers. Included research studies were evaluated to confirm that they were not a multiple report of an identical study by ensuring contrasting author names, observing geographical location, sample sizes and comparing interventions and outcomes.

## Quality appraisal

Quality was assessed by employing *The Effective Public Health Practice Project Quality Assessment Tool for Quantitative Studies* (2004) due to its strong content and test-retest reliability, and a capability for assessing diverse quantitative design approaches (Thomas et al., 2004). Quality is assessed by examining and rating (‘strong’, ‘moderate’ or ‘weak’) of: selection bias, study design, confounders, masking, data collection methods and withdrawals and drop-outs. From individual ratings, an overall global rating is ascertained. Intervention integrity, and analysis of allocation, unit of analysis and statistical methods are also required for intervention appraisal.

## Results

A search of the databases provided 785 results, with 132 of these being duplicates that were excluded. 553 were then eliminated after title and abstract reviewing as they did not meet the inclusion criteria of BCA intervention outcome (519), or age (5), with further journals discarded due to being protocols (8), reviews (17), or only utilising a qualitative methodology (4). The full text of the remaining journals was examined, with a further 95 excluded due to not having a BCA intervention outcome (9), age not within the range of 18–50 years (84) and the application of a qualitative methodology and analysis (2). Five journal articles were assessed as having met the criteria. See Supplement 3 for PRISMA flow diagram.

## Description of studies and results

A summary of details can be found in Table 1.

**Table 1.** Study characteristics and results.

Reference	Sample size, age and location	Study design and intervention description	Measure of BCA	Time point studied	Analysis conducted	Findings
Labrague et al. (2021)	Total: 128 Intervention group: 64 Control group: 64 18–49 years Philippines	Experimental RCT Effects of mobile text messaging. Control: No SMS. Provided with country’s health agencies brochures or pamphlets on BCA.	Breast Cancer and Heredity Knowledge Scale. Breast Self-Examination Knowledge Scale. Breast Self-Examination Self-Efficacy Scale – a subscale derived from Champion’s HBM Scale (1993). Frequency of Breast Self-Examination Scale.	Pre intervention and after 1 month of intervention.	*T*-test and ANCOVA	BCA knowledge *p* = 0.001*, BSE knowledge *p* = 0.010*, BSE self-efficacy *p* = 0.232, BSE frequency *p* = 0.69.
Sarker et al. (2022)	Total: 400 18–26 years Bangladesh	Quasi-experimental Pre-post study Health education intervention No control group	BCA knowledge assessed by items on symptoms, risk factors, treatment, prevention, screening and BSE.	Pre-intervention and 15 days post- intervention.	McNemar and paired sample *t*-tests	Breast cancer symptoms *p* < 0.001*, risk factors *p* < 0.001*, treatment <0.001*, prevention *p* < 0.001*, breast cancer screening *p* < 0.001*, process of BSE p < 0.001*, change in BSE practices *p* < 0.001*.
Occa and Suggs (2016)	Total: 194 Control: 25 Narr. video: 43 Did. video: 42 Narr. info 41 Did. info: 43 18–30 years Switzerland and Italy	Experimental, RCT Effects of different cancer communications Four treatment groups: 1. Narrative video 2. Didactic video 3. Didactic infographic 4. Narrative infographic Control: No exposure to cancer communication.	Effects of communication on BCA, BC diagnostic exams, BSE attitudes, BC screening through BSE.	In one session.	*T*-test and ANCOVA	Intervention effects (ANCOVA): Awareness *p* = 0.006*, knowledge of diagnostic exams *p* = 0.111, attitudes towards breast self-exam *p* = 0.009*, intentions to detect breast cancer early *p* = 0.016*. Didactic videos: Effective for awareness and knowledge. Narrative videos: Effective for attitudes towards breast self-exam and intention to perform it.
Alameer et al. (2019)	Total: 150 Intervention: 75 Control: 75 Intervention group: 39.03 ± 4.96 Control group: 38.97 ± 4.43 Saudi Arabia	Quasi-experimental pre-test-post-test design. Health education Control: Pamphlets that included general information about BC	Modified versions of: Breast Cancer Knowledge test (McCance et al., 1990). BSE practices (Champion, 1993) CBE and mammography practices (de Oliveira et al., 2018; Wang et al., 2012 ).	Pre-intervention, and post-intervention at 6 weeks and 3 months.	Independent *t*-test, chi-squared test, Mann-Whitney *U* test, Wilcoxon signed-rank test, McNemar’s test, Friedman test, logistic regression.	Knowledge scores between groups 6 weeks and 3 months post-intervention *p* < 0.001*. Knowledge intervention group increased *p* *<* 0.001 < *. BSE scores between groups 6 weeks and 3 months post-intervention *p* < 0.001*. BSE increased in intervention group *p* < 0.001*. CBE and mammography practice at 6 weeks and 3 months post intervention *p* < 0.001*.
Yi and Park (2012)	Total: 103* *22 completed entire intervention. 20–40 years. Korea	A pre-test and post-test quasi-experimental design. Breast health class delivered by trained breast cancer survivors No control group	Knowledge of Breast Cancer and BSE questionnaire, and self-efficacy (Choi, 1996 ). BSE skills and compliance.	Pre-intervention, 1 and 3 months post-education.	*T*-tests Friedman test and Wilcoxon-signed ranks test.	Knowledge *p* < 0.001*. BSE performance skills *p* < 0.001*. BSE compliance *p* < 0.001*. Self-efficacy *p* < 0.001*. No difference found between 1 and 3 months post-education in any of these components.

* Indicates significant result *p* < 0.05.

### Methods

Two RCT experimental studies (Labrague et al., 2021; Occa and Suggs, 2016) and three quasi-experimental of pre-test post-test design (Alameer et al., 2019; Sarker et al., 2022; Yi and Park, 2012) with varying durations in total of intervention analyses taken directly after the session (Occa and Suggs, 2016), 15 days (Sarker et al., 2022), 1 month (Labrague et al., 2021) and 3 months (Alameer et al., 2019; Yi and Park, 2012) post intervention were included.

### Participants

The included studies involved 894 participants in total that completed the interventions (total includes only the 22 participants that fully completed the intervention within Yi and Park’s (2012) study), of young women aged 18–50 years, with no limitation on setting or recruitment method. Sample sizes ranged from 22 to 400 participants.

### Intervention

Interventions occurred from 2012 to 2022 and varied in components and techniques used in examining the effect of these on BCA.

Labrague et al. (2021) examined the effect of mobile text messaging on BCA components based on social cognitive theory (Bandura, 1986). Intervention participants were sent 3–5 text messages each day for 1 month. BCA components examined were BSE knowledge, frequency and self-efficacy, and BCA knowledge. The control group did not receive any text messages; instead they were provided with the country’s health agencies’ BCA brochures and pamphlets. Occa and Suggs’ (2016) RCT examined the effect of communication types on BCA with four treatment groups consisting of videos utilising a narrative approach with the communication of a breast cancer patient’s story about discovering a breast lump after performing BSE, and a didactic approach with physician presented information. Both the narrative and didactic approaches communicated similar texts of the same quality and included the same actress. The third treatment group utilised a didactic infographic, with the fourth treatment group being presented with a narrative infographic. Both infographics contained the same key information as in the video and used the same colour themes. However, the narrative infographic also included a picture of the patient and two children that were also used in the narrative video. This infographic also contained a brief description of the actor’s breast cancer experience. The control group had no exposure to cancer communication.

Within the quasi-experimental pre-test and post-test designs, all utilised a form of a health education intervention. Sarker et al. (2022) divided participants into groups of 10–15, and educated women on BCA of symptoms, risk factors, treatment, prevention and screening, using stepwise BSE process images, lectures and discussions. Respondents were encouraged to share and demonstrate what they had learnt. Alameer et al. (2019) examined a BCA intervention based on the health belief model (HBM; Rosenstock, 1974) within an education programme that included a PowerPoint presentation with pictures and videos, and a practical BSE session. Participants were given time to ask questions, and to discuss important barriers regarding BSE practice and visiting healthcare centres and clinics to undergo screening. A second group acted as a control group, whereby they only received general breast cancer information pamphlets. Yi and Park (2012) examined the effect of a breast health class based on self-efficacy theory derived from Bandura’s (1986) social cognitive theory utilising verbal persuasion and vicarious experience through audio-visual presentations with breast cancer and BSE facts. Breast silicone models, wooden bead necklaces with varying bead sizes to represent different lump sizes, and also an information brochure and animations were utilised. Self-examination was encouraged during the session. The educators were breast cancer survivors that shared their breast cancer experiences to raise awareness and overcome stigma. All participants underwent the intervention, with no control group.

Interventions ranged in geographical location: Philippines (Labrague et al., 2021), Bangladesh (Sarker et al., 2022), Switzerland and Italy (bordering communities; Occa and Suggs, 2016), Saudi Arabia (Alameer et al., 2019) and Korea (Yi and Park, 2012).

### Outcomes

The primary outcome assessed was BCA which comprised of all or some of the components of BCA including knowledge about breast cancer, risk, screening and BSE. However, it was communicated and analysed in a variety of manners, timepoints and questionnaire type.

Labrague et al. (2021) employed the Breast Cancer and Heredity Knowledge Scale (Ondrusek et al., 1999), Breast Self-Examination Knowledge Scale based on the American Cancer Society’s guidelines (Alkhasawneh et al., 2009), Breast Self-Examination Self-Efficacy Scale (a subscale from Champion’s HBM Scale (1993)) and a Frequency of Breast Self-Examination Scale. Sarker et al. (2022) examined knowledge of breast cancer and BSE practices utilising a questionnaire that was designed for the research focusing on symptoms, risk factors, treatment, prevention, screening and BSE. Occa and Suggs (2016) examined the effect of didactic and narrative video and infographic messaging on BCA, knowledge of breast cancer’s diagnostic exams, attitudes towards breast self-exam and intention to screen for breast cancer through a breast self-exam. BCA measured breast cancer, breast cancer examination, symptoms and risk factors knowledge. Knowledge about breast cancer exams was assessed through correct/incorrect answers, with BSE attitude measured using a semantic differential scale whereby individuals indicated the most suitable adjective that described their feelings towards BSE, and intentions to screen for breast cancer assessing intentions to ask for a given breast examination. All items were informed by previous studies (Braithwaite et al., 2005; Francis et al., 2004; McCaul et al., 2003;) and were translated into Italian. Alameer et al. (2019) used a questionnaire that assessed knowledge of breast cancer, screening tools and practice, based on the Breast Cancer Knowledge test (McCance et al., 1990), BSE practices based on Champion’s (1993) and mammography practices based on questionnaires utilised by de Oliveira et al. (2018), and Wang et al. (2012). Yi and Park (2012) employed the Knowledge of Breast Cancer and BSE questionnaire developed by Choi (1996), with items focusing on incidence, symptoms, high risk factors, mammography period, BSE period, BSE procedure and BSE self-efficacy. However, it should be noted that when searching for Choi’s (1996) Knowledge of Breast Cancer and BSE questionnaire, it emerged that this is an unpublished dissertation, and it could not be accessed.

Time points studied varied throughout the papers, from on the day with all parts including pre-test, intervention and post-test conducted (Occa and Suggs, 2016), pre-intervention and 1 month after intervention commencement (Labrague et al., 2021), pre-intervention and 15 days after intervention (Sarker et al., 2022), across three time points of pre-intervention, 6 weeks and 3 months (Alameer et al., 2019), and 1 and 3 months post-intervention (Yi and Park, 2012). No study included costs.

## Intervention effects

All interventions showed positive BCA effects despite differing intervention methods and BCA component targets. Within the two RCTs, Labrague et al. (2021) demonstrated a significant increase in specific components by mobile text messaging of 3–5 SMS per day to improve breast cancer, and BSE knowledge, BSE frequency and BSE self-efficacy within the intervention group compared to the control group. BCA knowledge (*p* = 0.001) and BSE knowledge (*p* = 0.010) significantly increased. However, BSE self-efficacy, and BSE frequency were not found to significantly differ between the groups. Within Occa and Suggs’ (2016) examination of differing modes of BCA communication of narrative and didactic methods within infographics and videos, each mode demonstrated positive changes in BCA components. Videos demonstrated the greatest differences, but in itself results varied. Didactic was more effective for awareness and knowledge, and narrative for influencing attitudes towards breast self-exam and intention to perform it. One-way ANCOVAs compared intervention effects with significant results for awareness (*p* = 0.006), attitudes towards breast self-exam (*p* = 0.009) and intentions to detect breast cancer early (*p* = 0.016). However, no signiﬁcant differences were found between the intervention groups for diagnostic exams knowledge.

The three quasi-experimental studies that utilised health education formats of classroom-type education also showed similar findings. Sarker et al. (2022) reported significant increases of BCA knowledge within its educational intervention group among female students. These increases were found within knowledge of breast cancer symptoms (*p* < 0.001), risk factors (*p* < 0.001), treatment (*p* < 0.001), prevention (*p* < 0.001), breast cancer screening (*p* < 0.001), process of BSE (*p* < 0.001) and change in BSE practices (*p* < 0.001). Alameer et al. (2019) also reported effects within their educational intervention based on the HBM, with a significant increase in knowledge (*p* < 0.001), and BSE scores (*p* < 0.001) in the intervention group post intervention, with significant differences in knowledge (*p* < 0.001) and BSE (*p* < 0.001) scores between groups 6 weeks and 3 months post intervention. Clinical breast examination (CBE) and mammography practice scores demonstrated significant differences at 6 weeks and 3 months post intervention *p* < 0.001*.

Yi and Park’s (2012) breast examination class delivered by trained breast cancer survivors reported a significant difference in knowledge scores (*p* < 0.001), BSE performance skills scores (*p* < 0.001) and BSE performance compliance and regularity (*p* < 0.001). However, no significant differences were found within these components of BCA between 1 and 3 months post-education.

### Selection bias

Within the interventions assessed, it is not reported what percentage of individuals agreed to participate. It is therefore not included within this section rating.

Alameer et al.’s (2019) recruitment of female teachers came from eight schools that were randomly selected within a city, with the first four schools non-randomly allocated to the intervention group, and the remaining four to the control group. The evidence within this research does not indicate any selection bias issues with individuals very likely to be representative of the target population. Therefore, this research is rated as *strong* within this component.

Participants within Labrague et al.’s (2021) research were randomly selected from two communities, with those included very likely to be representative of the target population. Participants that met the eligibility criteria were randomised to either the intervention or control group. A rating of *strong* is therefore assigned.

Sarker et al. (2022) conducted a proportionate stratified random sampling technique within a specified target population of female university students for study sample size that is likely to be representative, however, only one university was recruited from. Overall, this indicates a *moderate* rating.

Occa and Suggs (2016) communicated that participants were recruited by two differing methods for feasibility reasons, with students at a university in Switzerland recruited through face-to-face methods by the paper’s lead author by directly approaching individuals and inviting them to participate. Participants in Italy undertook the intervention online and were recruited using a snowball sampling technique, with personal contacts of the lead author being directly invited through a direct Facebook message, with those approached asked to invite others or to provide names of interested individuals to the lead author. Despite the more direct nature of the research recruitment strategies that places participants as somewhat likely of the target population, those that agreed to participate in both groups were randomly assigned to one of the five groups. Overall, this research is rated as *moderate.*

Yi and Park (2012) advertised within a variety of institutions including community health clinics, colleges, private companies and social groups, with six BCA health interventions performed in community health clinics, three in private companies, one at a college and one in a social group. There was no random selection indicated as there was no control group utilised. It is also recognised that the attendance within the intervention of those within private companies was communicated as mandatory. This potentially affects the likelihood of participants being representative of the target population. Overall, this section for this research is rated as *weak.*

### Study design

Two of the studies assessed were classified as experimental RCTs by the papers’ authors. The method of randomisation was clearly described within Labrague et al.’s (2021) research with an appropriate method of randomisation performed using a computer-generated block randomisation (allocation ratio of 1:1) and a permuted block design (block size of 2–4). The study design is therefore rated as *strong.* However, whilst Occa and Suggs (2016) report that participants were randomly assigned to one of the five groups (4 intervention type, 1 control group), the method of randomisation is not communicated, therefore demonstrating characteristics of a CCT, rather than an RCT. Nevertheless, this still gives the study design a *strong* rating as per the quality assessment tool.

The remaining three research studies are quasi-experimental in their approaches, with two as a cohort one group pre and post intervention design (Sarker et al., 2022; Yi and Park, 2012), and one as a cohort analytic type with a two group pre and post design study (Alameer et al., 2019). These are therefore rated as *moderate.*

### Confounders

From the evidence provided, groups within the target populations were homogenous. Each intervention displayed sociodemographic information such as SES, age, education and marital status/family. From this perspective each intervention is rated as *strong*.

### Masking of experimental condition

As these interventions were to examine the effect on BCA, outcomes would have been communicated to participants. It is therefore not possible to be certain whether the outcome assessor(s) was (were) aware of the intervention status of participants within those that had more than one group, or whether the study participants were entirely aware of the research question. Overall, this component is rated as *weak.*

### Data collection methods

All studies utilised self-reported data within survey and questionnaire methods demonstrating either a ‘face’ validity or ‘content’ validity, or both. Data collection methods were outlined, detailing components that were examined. Therefore, a *strong* rating is awarded.

Labrague et al. (2021) report that scales were translated to the local language (Filipino) using forward and backward translation. To ensure face validity, two experts in the Filipino and English language fields with a nursing education were consulted. From these translations and modifications, the Cronbach’s α of the breast cancer knowledge scale was 0.88, the breast self-examination scale was also 0.88, breast self-examination self-efficacy scale was 0.90 and the frequency of breast self-examination scale was 0.88.

Sarker et al. (2022) assessed breast cancer knowledge by designing and focusing measures on symptoms, risk factors, treatment, prevention, screening, BSE process and BSE practice. The content breakdown of the measure demonstrates content validity within the collected self-reported data. Alameer et al.’s (2019) communication of the adaptation of previously utilised measures within this study area, also provides validity, with researcher designed measurements such as Yi and Park’s (2012) BSE proficiency test openly communicated. Despite being unable to source the original measurement used by Yi and Park (2012) of Choi (1996), items and component focus are communicated for breast cancer and BSE knowledge, and self-efficacy.

Occa and Suggs (2016) examination of the effects of communication on BCA components was undertaken by analysing four outcome measures of BCA, knowledge of breast cancer’s diagnostic exams, intention to screen for breast cancer through a breast self-exam, and attitudes towards breast self-exam. The items were informed by previous studies that were conducted in English on breast cancer communication and were subsequently translated into Italian. The authors have therefore demonstrated both face validity and content validity.

### Withdrawals and drop-outs

Alameer et al. (2019) demonstrated evidence of 80%–100% participation (loss of *N* = 1 within the intervention group and *N* = 2 within the control group at 3 months post-intervention). At the 6-week data collection point there was a 100% response rate in both groups. The 3-month data collection point had response rates of 98.7% and 97.3% in the intervention and control groups respectively. Loss of participation numbers was reported as due to either the inability to contact the participant, or refusal by an individual to participate any further. Overall, a *strong* rating is conferred.

Sarker et al.’s (2022) analyses indicate that there was a high participation level, with no loss in participant numbers throughout, demonstrating a *strong* rating.

Occa and Suggs (2016) indicated that four ineligible individuals were removed, however the reasons were not communicated. Nevertheless, this indicates a high level of completion at 80%–100%, giving the research a *strong* rating.

Labrague et al. (2021) did not indicate any withdrawal and drop-out numbers within the analysis which given the 1-month timeframe of a mobile intervention deems this a *weak* rating.

Yi and Park’s (2012) research indicated a low percentage of participants completing the study of less than 60%. Despite a total of 103 agreeing to participate within the intervention, only 22 individuals responded both at 1 and 3 months post-education (response rate 21.36%), with the reason given of not returning questionnaires at the time points required. This, therefore, gives this section a *weak* rating.

### Intervention integrity

Within Labrague et al.’s (2021) intervention, there was an equal distribution between the intervention group (50%) and control group (50%). Each participant within the intervention group received the same mobile text messaging, with control group participants instead receiving BCA brochures and pamphlets. However, it is not possible to ascertain how intervention consistency was measured, if at all, and the likelihood that subjects received an unintended intervention that may influence the results. For example, it is unclear what particular differences there are, if any, between the information contained within the brochures given to the control group, and the BCA messages that were sent to those within the experimental group.

All of Sarker et al.’s (2022) participants (100%) received the intervention due to this being a cohort one group pre- and post-test design. Consistency of the intervention is unclear; however, it is noted that all sessions were set in the respondents’ dormitories, with similar group sizes (10–15), and sessions run in similar formats to each other. Again, it is also unclear of the likelihood that subjects received an unintended intervention caused by contamination or co-intervention within the teaching times, or outside these.

The participants within Occa and Suggs (2016) intervention were divided into five groups, four being experimental, and one being a control as follows; control group (*N* = 25, 12.89%), narrative video (*N* = 43, 22.17%), didactic video (*N* = 42, 21.65%), narrative infographic (*N* = 41, 21.13%) and didactic infographic (*N* = 43, 22.17%). Groups were described as homogeneous. While the participant numbers within experimental groups are evenly distributed, the control group has considerably less individuals, which may affect outcomes. It is not possible to report if consistency of intervention was measured, or if subjects received an unintended intervention that may influence results.

There was equal distribution of participants within Alameer et al.’s intervention (50% intervention group and 50% control group). Again, there is no inclusion of details on consistency of intervention, or potential contamination or co-intervention at any of the time points studied (baseline, 6 weeks or 3 months).

While Yi and Park (2012) reported the lowest level of participation (21.36%), all 103 individuals initially partook in the BCA education intervention due to the nature of the cohort design. It is uncertain how, if at all, intervention consistency was measured, or the likelihood of participants receiving an unintended intervention.

### Analyses

It is considered that appropriate analyses have occurred in each intervention, within the unit of allocation, and unit of analysis. The unit of analysis was each individual woman having been exposed to either an intervention to increase BCA, or to a control group. Utilising the units of allocation terminology provided by the quality assessment tool as denoted within brackets, the unit of allocation varied across interventions. These are from the community (community; Labrague et al., 2021), university (organisation/institution; Sarker et al., 2022), university and Facebook (organisation/institution, and community; Occa and Suggs, 2016), schools (organisation/institution; Alameer et al., 2019), and via a variety of units within Yi and Park’s (2012) intervention, including community health clinics, private companies, college and social group (community, organisation/institution and practice/office).

### Summarisation

A global rating for each study is awarded as follows: *strong* (no weak ratings), *moderate* (one weak rating) and *weak* (two or more weak ratings). No papers demonstrate a *strong* study quality, with Sarker et al. (2022), Occa and Suggs (2016) and Alameer et al. (2019) indicating a *moderate* study quality. Labrague et al. (2021) and Yi and Park (2012) demonstrate a *weak* study. The overall rating is deemed as *weak.*

## Discussion

This systematic review evaluated the effect of BCA interventions on young women’s (aged 18–50 years) BCA knowledge levels. Five studies were reviewed. Two studies were experimental in design, with three utilising a quasi-experimental approach with pre-and post-test analysis. All studies occurred within the last 10 years of when the systematic review was commenced in 2022. Overall, the evidence is weak in investigating the effectiveness of BCA interventions on this cohort and is considered as inconclusive.

All interventions demonstrated significant positive changes within BCA components, which is comparable to a previous systematic review investigating BCA and screening uptake via public health campaigns and educational interventions within the UK (Anastasi and Lusher, 2019). However, this previous review also narrated a wide variety of methods and settings, highlighting the difficulty experienced in evaluating BCA interventions in a conclusive manner. Within this systematic review, significant positive effects on BCA levels were found within mobile text messaging (Labrague et al., 2021), health education interventions (Alameer et al., 2019; Sarker et al., 2022; Yi and Park, 2012), and by applying various communication methods within narrative and didactic modes (Occa and Suggs, 2016). Only one valid RCT was found (Labrague et al., 2021) by clear communication of a randomisation method, with significant results across several components including BCA, and BSE knowledge. However, BSE self-efficacy, and BSE frequency were not found to significantly increase at the time point of analysis at 1-month post intervention commencement. This is noteworthy when considering the design of a BCA intervention for young women. With self-efficacy being an individual’s perceived confidence in their capability to perform a behaviour (Bandura, 1977), in this case BCA, this may negatively affect a person’s level of effort or persistence in maintaining behaviours, despite being educated on BCA components.

## Strengths and limitations

This systematic review carries limitations, primarily that of the variation in study quality. Certain aspects such as Yi and Park’s (2012) low percentage of participant completion create a difficulty in making a robust conclusion despite significant positive findings pertaining to knowledge, BSE skills and performance and self-efficacy. Its finding of no significant differences between 1 and 3 months post-education is problematic to appraise as a consequence of the low response rate.

Randomisation was adequate within one of the two experimental studies. Both studies of Labrague et al. (2021) and Occa and Suggs (2016) claimed to be RCTs, however only Labrague et al.’s (2021) intervention communicated its randomisation method. Within Occa and Suggs (2016) research, the method of randomisation is not communicated which depicts it as a CCT, rather than a RCT. However, this method is still preferable to the remaining three studies within the review as per the *Effective Public Health Practice Project’s Quality Assessment Tool for Quantitative Studies* (2004) that have utilised a quasi-experimental design, with two as a cohort one group pre and post intervention design with no control group (Sarker et al., 2022; Yi and Park, 2012), and one as a cohort analytic type, with a two group pre and post design study (Alameer et al., 2019). Findings from experimental designs allow for increased confidence in outcomes that can contribute to potential implications to key stakeholders such as clinicians, individuals that design health behaviour interventions, and to the target audience of young women. RCTs represent a ‘gold standard’ in study design, predominantly for their ability to control for confounding factors (Sheikh et al., 2002).

Sample sizes and time periods broadly varied, with participant numbers ranging from *N* = 22 to *N* = 400, and from one session to 3 months post intervention making it problematic to derive any significant conclusions across all studies. Perhaps most critical to note from the outcomes of this review, is that the interventions themselves varied from mobile phone messaging (Labrague et al., 2021), communication methods (Occa and Suggs, 2016) and health education type interventions (Alameer et al., 2019; Sarker et al., 2022; Yi and Park, 2012). Despite three out of the five papers utilising health education methods, these also varied considerably in measurements utilised to capture BCA levels, setting, country and type of materials employed to affect levels of awareness. Future research should aim to utilise a standard measure of BCA that examines components such as the Breast-CAM (Linsell et al., 2010), focusing on key factors of risk, screening and self-examination. Varying measures create difficulties in ascertaining a clear conclusion when examining intervention effects. Despite there being a variety of countries represented, it is not extensive enough for a global representation of findings.

Utilising theory can increase intervention effectiveness (Glanz and Bishop, 2010). Whilst three cited theories; social cognitive theory (Labrague et al., 2021), self-efficacy theory (Yi and Park, 2012) and HBM (Alameer et al., 2019), and one recognised cognitive and heuristic aspects (Occa and Suggs, 2016), another did not apply theory (Sarker et al., 2022) despite exploring knowledge, attitudes and practices. In many cases, interventions are designed and based on implicit common-sense behaviour models, without evidence of theory and formal target behaviour analysis (Davies et al., 2010). No studies demonstrated a well-mapped intervention with considered components of a behaviour change theory which may also explain differences.

While masking is considered important particularly when conducting research into the efficacy of new interventions (Sheikh et al., 2002), no research within this review undertook masking. However, due to it evident that BCA was being targeted, it is difficult to achieve this for outcome assessors and participants which may contribute to experimental bias.

### Review level

This systematic review solely included English-language publications and engaged with seven databases; Cochrane Library (CENTRAL), APA PsycArticles, APA PsycInfo, MEDLINE, ASSIA, CINAHL and Web of Science. Therefore, other publications may have been omitted, however, there were 132 duplicates found within a total of 785 results, demonstrating that this search was satisfactory and inclusive.

The search terms and Boolean operators employed, and the inclusion and exclusion criteria imposed may have also reduced potential outcome numbers. However, previous systematic reviews concerning BCA such as those conducted by Anastasi and Lusher (2019) and O’Mahony et al. (2017) were referred to, to examine terms. From the research that has been undertaken for topic familiarisation, this also contributed to word choices and phrases.

Nevertheless, it is acknowledged that the review was primarily undertaken by one individual, with Cochrane advising that systematic reviews should be performed by several individuals (Lasserson et al., 2024). It is recognised that researcher bias may have influenced outcomes.

## Conclusions

From recognising the difficulty in generating concrete conclusions, a reasonable suggestion for future research would be to adopt a greater standardised approach with measures (e.g. Breast-CAM; Linsell et al., 2010). Espousing more ‘gold standard’ experimental study designs such as RCTs or CCTs if complete blind randomisation is not possible will also foster enhanced findings for improved communication of implications to both clinicians and patients.

Positive outcomes across all intervention designs may infer that young women under the age of 50 years may be receptive to a variety of engaging designs. However, the small number of studies highlights a dearth of BCA interventions specific to young women, with often a stereotype association that breast cancer is for older ages, overlooking diverse social, personal and medical challenges that differ to women who develop breast cancer at a later age (Costa et al., 2024).

Recent developments highlight the importance of categorising intervention components and mapping these directly to change mechanisms (e.g. see Michie et al., 2014). A more systematic and targeted approach with proper application and understanding of health behaviour change mechanisms is warranted.

## Supplemental Material

sj-docx-1-hpq-10.1177_13591053241270614 – Supplemental material for The effect of breast cancer awareness interventions on young women aged 18–50 years: A systematic review Supplemental material, sj-docx-1-hpq-10.1177_13591053241270614 for The effect of breast cancer awareness interventions on young women aged 18–50 years: A systematic review by Caitríona Plunkett, Melissa Pilkington and Joseph Keenan in Journal of Health Psychology
