# Supplementary material for: The effect of breast cancer awareness interventions on young women aged 18–50 years: A systematic review
Source: J Health Psychol. 2024 Aug 12;30(3):559–75. doi: 10.1177/13591053241270614 (PMC11894831; doi:10.1177/13591053241270614)

**Supplemental material:**

Supplementary 1

*Set Limits Within Databases*

| **Database** | **Limits** |
| --- | --- |
| **Medline (through PubMed)**    Results: 33 | Case reports, Clinical study, Observational study, Randomised controlled trial, in the last 10 years, English, Female, Medline. |
| **APA Psychinfo**    Results: 101 | Publication year 2012-2022, Academic journals and dissertations, English, Adulthood (18 years and older), Quantitative. |
| **APA PsychArticles** | Publication year 2012-2022, All journals, English, Adulthood (18 years and older), Quantitative. |
| **Web of Science** | 2012 -2022, Open Access and Early Access, Document types: Article and early access, English. |
| **Cochrane Library** | 2012-2022, trials. |
| **ASSIA** | 2012-2022, English, Scholarly journals, dissertations, and theses. Article, English. |
| **CINAHL** | 2012-2022, Female, All adult, Academic journals, English. |

Supplementary 2

*MEDLINE MESH SEARCH*

| MEDLINE |
| --- |
| (("breast cancer aware*"[All Fields] OR "breast aware*"[All Fields] OR "breast self exam*"[All Fields] OR "breast self exam*"[All Fields]) AND ("intervention s"[All Fields] OR "interventions"[All Fields] OR "interventive"[All Fields] OR "methods"[MeSH Terms] OR "methods"[All Fields] OR "intervention"[All Fields] OR "interventional"[All Fields] OR ("program"[All Fields] OR "program s"[All Fields] OR "programe"[All Fields] OR "programed"[All Fields] OR "programes"[All Fields] OR "programing"[All Fields] OR "programmability"[All Fields] OR "programmable"[All Fields] OR "programmably"[All Fields] OR "programme"[All Fields] OR "programme s"[All Fields] OR "programmed"[All Fields] OR "programmer"[All Fields] OR "programmer s"[All Fields] OR "programmers"[All Fields] OR "programmes"[All Fields] OR "programming"[All Fields] OR "programmings"[All Fields] OR "programs"[All Fields]) OR ("program"[All Fields] OR "program s"[All Fields] OR "programe"[All Fields] OR "programed"[All Fields] OR "programes"[All Fields] OR "programing"[All Fields] OR "programmability"[All Fields] OR "programmable"[All Fields] OR "programmably"[All Fields] OR "programme"[All Fields] OR "programme s"[All Fields] OR "programmed"[All Fields] OR "programmer"[All Fields] OR "programmer s"[All Fields] OR "programmers"[All Fields] OR "programmes"[All Fields] OR "programming"[All Fields] OR "programmings"[All Fields] OR "programs"[All Fields]) OR "educat*"[All Fields] OR "promot*"[All Fields]) AND ("womans"[All Fields] OR "women"[MeSH Terms] OR "women"[All Fields] OR "woman"[All Fields] OR "women s"[All Fields] OR "womens"[All Fields] OR ("womans"[All Fields] OR "women"[MeSH Terms] OR "women"[All Fields] OR "woman"[All Fields] OR "women s"[All Fields] OR "womens"[All Fields]) OR "young woman"[All Fields] OR "young women"[All Fields] OR ("student s"[All Fields] OR "students"[MeSH Terms] OR "students"[All Fields] OR "student"[All Fields] OR "students s"[All Fields]))) AND ((y_10[Filter]) AND (casereports[Filter] OR clinicalstudy[Filter] OR clinicaltrial[Filter] OR observationalstudy[Filter] OR randomizedcontrolledtrial[Filter]) AND (female[Filter]) AND (medline[Filter]) AND (english[Filter])) |

Supplementary 3

*PRISMA (2009) Flow Diagram*


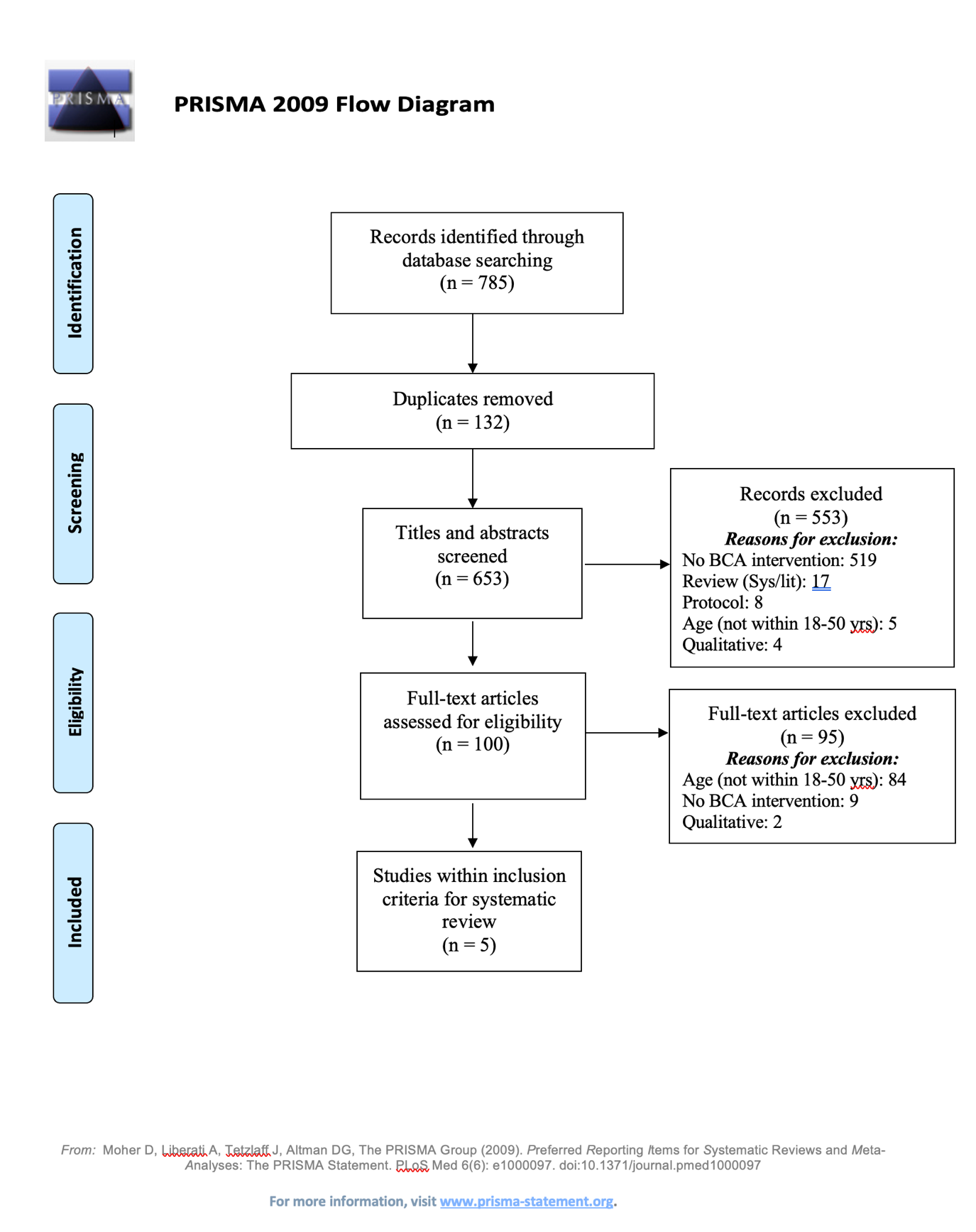

Supplement: sj-docx-1-hpq-10.1177_13591053241270614 – Supplemental material for The effect of breast cancer awareness interventions on young women aged 18–50 years: A systematic review [file sj-docx-1-hpq-10.1177_13591053241270614.docx]
